# Therapeutic Benefit of Radial Optic Neurotomy in a Rat Model of Glaucoma

**DOI:** 10.1371/journal.pone.0034574

**Published:** 2012-03-29

**Authors:** Nicolás Belforte, Pablo H. Sande, Nuria de Zavalía, Damián Dorfman, Ruth E. Rosenstein

**Affiliations:** Laboratory of Retinal Neurochemistry and Experimental Ophthalmology, Department of Human Biochemistry, School of Medicine/CEFyBO, University of Buenos Aires, CONICET, Buenos Aires, Argentina; Charité University Medicine Berlin, Germany

## Abstract

Radial optic neurotomy (RON) has been proposed as a surgical treatment to alleviate the neurovascular compression and to improve the venous outflow in patients with central retinal vein occlusion. Glaucoma is characterized by specific visual field defects due to the loss of retinal ganglion cells and damage to the optic nerve head (ONH). One of the clinical hallmarks of glaucomatous neuropathy is the excavation of the ONH. The aim of this work was to analyze the effect of RON in an experimental model of glaucoma in rats induced by intracameral injections of chondroitin sulfate (CS). For this purpose, *Wistar* rats were bilaterally injected with vehicle or CS in the eye anterior chamber, once a week, for 10 weeks. At 3 or 6 weeks of a treatment with vehicle or CS, RON was performed by a single incision in the edge of the neuro-retinal ring at the nasal hemisphere of the optic disk in one eye, while the contralateral eye was submitted to a sham procedure. Electroretinograms (ERGs) were registered under scotopic conditions and visual evoked potentials (VEPs) were registered with skull-implanted electrodes. Retinal and optic nerve morphology was examined by optical microscopy. RON did not affect the ocular hypertension induced by CS. In eyes injected with CS, a significant decrease of retinal (ERG a- and b-wave amplitude) and visual pathway (VEP N2-P2 component amplitude) function was observed, whereas RON reduced these functional alterations in hypertensive eyes. Moreover, a significant loss of cells in the ganglion cell layer, and Thy-1-, NeuN- and Brn3a- positive cells was observed in eyes injected with CS, whereas RON significantly preserved these parameters. In addition, RON preserved the optic nerve structure in eyes with chronic ocular hypertension. These results indicate that RON reduces functional and histological alterations induced by experimental chronic ocular hypertension.

## Introduction

Glaucoma is a leading cause of blindness worldwide, characterized by specific visual field defects due to the degeneration of retinal ganglion cells (RGCs) and damage to the optic nerve head (ONH). Elevated intraocular pressure (IOP) is the most important risk factor for the development of glaucoma. However, the underlying mechanisms that link elevated IOP to RGC death are still not fully understood. An experimental model of pressure-induced optic nerve damage would facilitate the understanding of the cellular events leading to RGC death, and how they are influenced by IOP and other risk factors. Recently, we have developed a new model of glaucoma in rats through weekly injections of chondrotin sulfate (CS) in the eye anterior chamber. Acute or chronic intracameral injections of CS significantly increase IOP as compared with vehicle-injected eyes [1]. Moreover, injections of CS for 6 or 10 (but not 3) weeks significantly decrease electroretinographic activity as well as flash visual evoked potentials (VEPs). After 10 weeks of ocular hypertension induced by CS, a significant loss of RGCs and optic nerve fibers occurs in CS-treated eyes [1]. These results indicate that weekly intracameral injections of CS mimic central features of human primary open-angle glaucoma. Thus, this model could be a useful tool for understanding the pathogenic mechanisms involved in glaucomatous neuropathy, as well as for the development of new therapeutic strategies.

One of the clinical hallmarks of glaucomatous optic neuropathy is the excavation of the ONH, which consists in a progressive posterior displacement of the ONH surface and excavation of the prelaminar tissues beneath the anterior most aspect of the scleral canal, the anterior scleral ring [2], [3]. A considerable body of literature characterized the classic posterior bowing and compression of the lamina cribrosa and excavation of the scleral canal wall beneath the opening in Bruch's membrane in moderately and severely damaged glaucomatous eyes [4], [5]. Both plastic (permanent) and hypercompliant deformations of the lamina cribrosa and anterior scleral canal wall were described in young adult monkey eyes with early experimental glaucoma [6] which have been verified by three-dimensional reconstructions of serially sectioned ONH and peripapillary sclera [7]. It was postulated that damage to the ONH, the lamina cribrosa, and anterior scleral canal wall connective tissue plays a key role in glaucomatous neuropathy [8], [9].

Central retinal vein occlusion (CRVO) is a compartment-like syndrome resulting from increased pressure on the central retinal vein (CRV) within the confined space of the scleral ring which results in tissue ischemia. In 2001, Opremcak and coworkers proposed radial optic neurotomy (RON), in which a microvitreoretinal blade is used to cut the cribriform plate, the scleral ring, and the adjacent sclera in a radial fashion, to alleviate the constriction of the scleral outlet, as a new surgical option for CRVO [10]. In this pilot study, the authors reported that 8 of 11 patients showed an average improvement in visual acuity of five lines after a mean follow-up of 9 months, whereas only two patients worsened [10]. In 2006, the same group reported 117 cases treated with RON, in which a gain of two or more lines was observed for 78% of patients [11]. Improvements in visual acuity by RON have been confirmed by other investigators [12]–[15]. However, it is still under debate, whether RON would be an adequate treatment modality or a dangerous procedure with potentially severe complications [16]–[19]. Recently, we have shown that RON provokes only minor histological changes, and transient functional alterations in normal *Wistar* rat eyes [20].

Since the connective tissues of the anterior scleral canal wall are permanently deformed at early stages of glaucoma [6], and considering that the ONH connective tissues are exposed to substantial levels of IOP-related stress/strain [3], [21], [22], in the present study, we tested the hypothesis that by alleviating the constriction of the scleral outlet induced by ocular hypertension, RON could prevent and/or reduce experimental glaucomatous damage.

## Results

**Figure 1:**
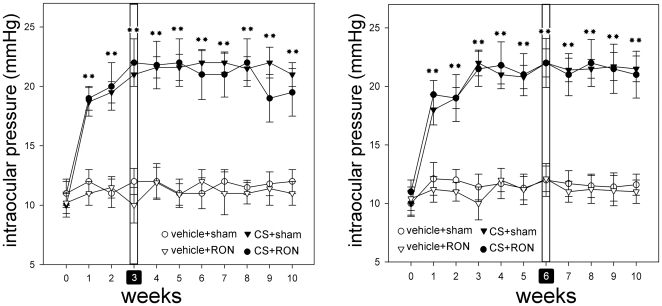
IOP in eyes injected with vehicle or CS with or without RON. TonoPen measurements of IOP from eyes bilaterally injected with vehicle or CS and submitted to a sham procedure or RON performed at 3 (left panel) or 6 (right panel) weeks of treatment with vehicle or CS. At all time points examined, CS significantly increased IOP as compared with vehicle-injected eyes. RON did not modify this parameter in vehicle or CS-injected eyes at any time point. Data are the mean ± SEM (n = 10 eyes per group). **p<0.01 versus vehicle-injected eyes with sham procedure, by Tukey's test.

Figure 1 depicts the average IOP from eyes submitted to RON or sham procedure at 3 or 6 weeks of treatment with weekly injections of vehicle or CS. IOP was significantly higher in CS- than in vehicle-injected eyes, while sham operation or RON performed at 3 or 6 weeks of treatment did not modify IOP in both groups, at all the time points examined. No significant differences in IOP values were observed between intact eyes and eyes injected with vehicle and submitted to a sham procedure (data not shown).

**Figure 2:**
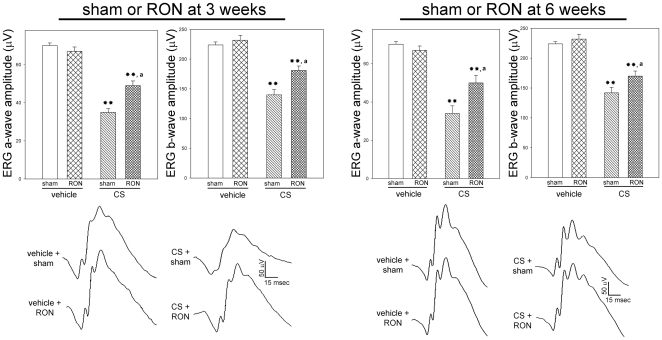
Electroretinographic preservation in hypertensive eyes induced by RON. ERGs were registered after 10 weeks of treatment with vehicle or CS in eyes submitted to sham operation or RON at 3 (left panel) or 6 weeks (right panel) of treatment. In sham operated eyes, CS induced a significant decrease in ERG a- and b-wave amplitude, as compared with vehicle-injected eyes. In hypertensive eyes submitted to RON at 3 or 6 weeks of ocular treatment with CS, a significant reduction of these alterations was observed. The lower panel shows representative scotopic ERG traces from eyes injected with vehicle or CS with or without RON. Data are the mean ± SEM (n = 10 eyes per group). **p<0.01 versus vehicle injected eyes with sham operation (sham); a: p<0.05 versus CS-injected eyes with sham operation, by Tukey's test.

**Figure 3:**
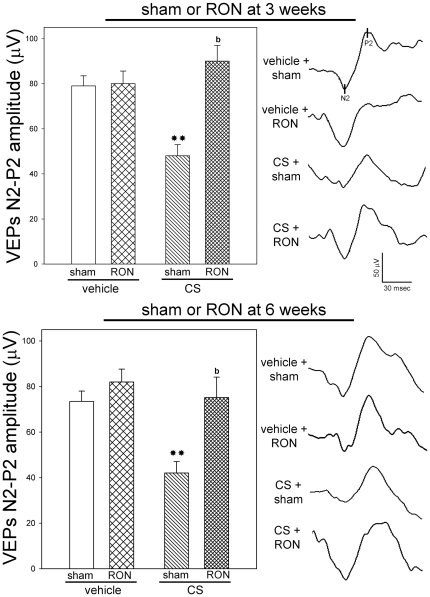
Flash VEPs in eyes injected with vehicle or CS with or without RON. Animals were weekly injected with vehicle or CS for 10 weeks and submitted to a sham operation or RON at 3 (upper panel) or 6 (lower panel) weeks of intracameral injections. A significant reduction in flash VEP N2-P2 amplitude component was observed in eyes injected with CS with a sham procedure. RON significantly abrogated the effect of ocular hypertension. No changes between vehicle- injected eyes with or without RON were observed. Representative VEPs traces are shown on the right side. Data are mean ± SEM (n = 10 eyes per group). **p<0.01 versus vehicle-injected eyes without RON (sham), b: p<0.01 versus CS-injected eyes with sham procedure (sham), by Tukey's test.

In order to assess the effect of RON on functional alterations induced by chronic ocular hypertension, the functional state of retinas from eyes weekly injected with vehicle or CS for 10 weeks with or without RON was analyzed by scotopic electroretinography. The average amplitude of scotopic electroretinogram (ERG) a- and b- waves of rats injected with vehicle or CS for 10 weeks and submitted to RON in one eye and a sham procedure in the contralateral eye at 3 (left panel) or 6 (right panel) weeks of ocular hypertension is depicted in Figure 2. In sham operated eyes, weekly injections of CS for 10 weeks significantly decreased scotopic ERG a- and b-wave amplitude. RON performed either 3 or 6 weeks after the onset of CS treatment significantly decreased the ERG dysfunction, as shown in Figure 2. Representative scotopic ERG traces from rats injected with vehicle or CS for 10 weeks with or without RON are also shown in Figure 2. To assess the visual pathway function, flash VEPs were registered at 10 weeks of treatment with vehicle or CS in eyes submitted to sham operation or RON. CS injections decreased the VEP N2-P2 component amplitude, while RON performed at 3 or 6 weeks of ocular hypertension significantly preserved this parameter (Figure 3). Representative VEP traces from all the groups are also shown in Figure 3. No noteworthy changes in ERG a- and b- wave, and VEP N2-P2 component latency were detected among experimental groups.

**Figure 4:**
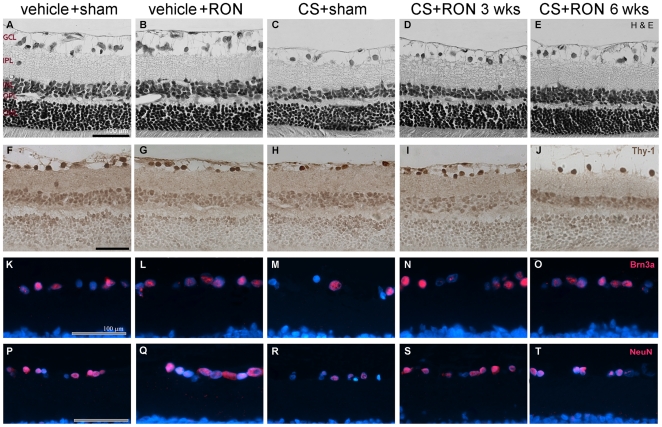
Retinal histology examination after 10 weeks of ocular hypertension. A–E: Representative photomicrographs of retinal sections stained with hematoxylin and eosin from a vehicle-injected sham operated eye at 3 weeks of intracameral treatment (A), a vehicle-injected eye submitted to RON at 3 weeks of intracameral treatment (B) and a hypertensive eye without (C) or with RON performed at 3 (D) or 6 (E) weeks of treatment with CS. Note the diminution of GCL cells in the eye injected with CS without RON. RON preserved this parameter. The other retinal layers showed a normal appearance in all groups. Immunohistochemical detection of Thy-1 (F–J), Brn3a (K–O) or NeuN (P–T)-positive cells in the GCL from a vehicle-injected eye submitted to a sham procedure or RON, a hypertensive eye without or with RON performed at 3 or 6 weeks of treatment. The presences of all these markers were confined to the GCL in all experimental groups. A decrease in GCL cell number was observed in CS- injected eyes with sham procedure as compared with vehicle-injected eyes (sham or RON), whereas RON, which showed no effect in vehicle-injected eyes, preserved GCL cell count in CS-injected eyes. No differences were observed between CS-injected eyes submitted to a sham operation at 3 and 6 weeks of treatment (not shown). Scale bar: 100 µm. A representative (out of five per group) photograph of retina is shown. GCL, ganglion cell layer; IPL, inner plexiform layer; INL, inner nuclear layer; OPL, outer plexiform layer; ONL, outer nuclear layer.

The effect of RON on retinal histological alterations induced by ocular hypertension was examined. A morphometric analysis of retinal sections performed at 10 weeks of treatment with vehicle or CS revealed no differences in the total retina, inner plexiform layer (IPL), inner nuclear layer (INL), outer plexiform layer (OPL), outer nuclear layer (ONL) thickness (data not shown), whereas a decrease in the number of cells in the ganglion cell layer (GCL) was observed in CS-treated eyes submitted to a sham procedure. RON reduced GCL cell loss, as shown in Figure 4 and Table 1. Thy-1, Brn3a and NeuN-positive cells in the retina from vehicle or CS-injected eyes with or without RON were counted. A statistically significant increase in the number of hematoxylin and eosin (H&E) stained cells, and in Thy-1, Brn3a and NeuN-positive cells in the GCL was observed in hypertensive eyes submitted RON at 3 or 6 weeks of ocular hypertension, as compared with hypertensive eyes submitted to a sham procedure (Figure 4, Table 1).

**Table 1:** Effect of RON on GCL cell count in vehicle or CS-injected eyes.

	sham operation or RON at 3 weeks			
	number of cells in GCL/100 µm			
	vehicle		CS	
	sham	RON	sham	RON
H&E	9,7±0,7	9,0±0.6	6,1±0,7**	9,9±0,3^b^
Thy-1	4,1±0,1	4,0±0,2	2,1±0,1**	4,2±0,1^b^
NeuN	4,6±0,5	4,2±0,4	1,8±0,4**	3,5±0,1^a^
Brn3a	3,1±0,1	2,9±0,2	1,7±0.1**	3,0±0.2^b^

	sham operation or RON at 6 weeks			
	number of cells in GCL/100 µm			
	vehicle		CS	
	sham	RON	sham	RON
H&E	9,5±0,6	9,2±0,5	5,0±0,2**	7,6±0,3*,^b^
Thy-1	4,0±0,3	3,8±0,3	2,0±0,2**	3,4±0,1^b^
NeuN	4,4±0,4	4,5±0,5	1,6±0,3**	3,2±0,1^a^
Brn3a	3,4±0,2	3,1±0,3	1,9±0,2**	3,0±0,3^a^

Cell count in the GCL/100 µm was evaluated by H&E staining and Thy-1, NeuN and Brn3a immunostaining after 10 weeks of treatment with vehicle or CS in eyes submitted to sham operation or RON at 3 (upper panel) or 6 (lower panel) weeks of treatment. In sham operated eyes, CS induced a significant decrease in the GCL cell and in Thy-1, NeuN and Brn3a positive ganglion cell number, whereas in hypertensive eyes submitted to RON at 3 or 6 weeks, a significant preservation of GCL cells was observed. Data are the mean ± SEM (n = 5 retinas per group);

* p<0.05,

** p<0.01 versus vehicle- injected eyes with sham operation (sham);

a : p<0.05,

b : p<0.01 versus CS-injected eyes with sham operation, by Tukey's test.

**Figure 5:**
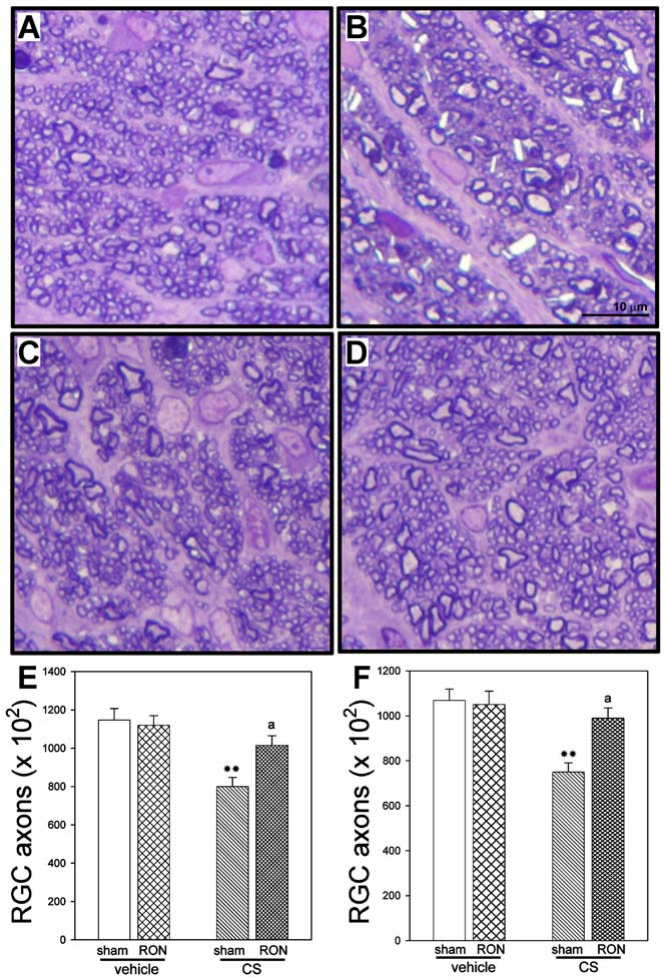
ON from a vehicle- or a CS-treated eye with or without RON. (A) Healthy, intact control optic nerve. Note the homogeneity of the staining. In vehicle-injected eyes, individual axons were generally uniform in shape, rounded and packed together tightly to form the fibers of the healthy nerve. In CS-treated eye with sham procedure (B) a less stained area indicates a nerve alteration. Disease in individual axons was characterized by axonal distention and distortion that resulted in a departure from the circular morphology of normal axons. In contrast, a conserved structure of the ON was observed in the CS-treated eye with RON at 3 (C), or 6 weeks (D) of ocular hypertension. Toluidine blue. Number of axons in eyes injected with vehicle or CS with sham procedure or RON at 3 (E) or 6 (F) weeks of treatment. A significant decrease in the axon number was observed in CS- injected eyes without RON as compared with vehicle-injected eyes (sham), whereas RON significantly preserved this parameter. Scale bar: 10 µm. Data are mean ± SEM (n = 5 eyes/group). **p<0.01 versus vehicle injected eyes with sham procedure (sham), a: p<0.05 versus CS-injected eyes without RON, by Tukey's test.

The optic nerve (ON) from eyes treated with CS for 10 weeks and submitted to sham procedure exhibited an overall loss of staining uniformity and integrity, showing distention and distortion that resulted in a departure from the circular morphology of normal axons. RON performed at 3 or 6 weeks of ocular hypertension significantly preserved ON structure (Figure 5). No foci of hemorrhage, interstitial edema, and inflammatory cells were observed in the ON from eyes submitted to RON. Moreover, chronic injections of CS induced a significant decrease in the axon number in sham operated eyes which was abrogated by RON performed at 3 or 6 weeks of treatment (Figure 5).

## Discussion

For the first time, the present results indicate that RON, which showed no effect *per se*, decreased functional and histological alterations induced by chronic ocular hypertension in the rat eye. Notably, the retinal protection induced by RON was independent from ocular hypertension, as shown by the fact that it did not affect the increase in IOP induced by CS injections.

Human primary open angle glaucoma is a progressive optic neuropathy. In the experimental model of glaucoma induced by weekly injections of CS, we have identified different stages, that show the following characteristics: i) 3 weeks of ocular hypertension: no changes in the ERG, VEPs, and retinal morphology (i.e. **asymptomatic ocular hypertension**); ii) 6 weeks of ocular hypertension: decrease in ERG and VEPs, without histological changes (i.e. **moderated glaucoma**); and iii) 10 weeks of ocular hypertension: further decrease in ERG and VEPs (vs. 6 weeks), and loss of RGCs and optic nerve fibers (i.e. **advanced glaucoma**) [1].

Although RON performed at 3 weeks of treatment conferred significant neuroprotection in the experimental model of glaucoma induced by CS, the translational relevance of this result is limited by the fact that the surgery was performed at a time point in which no functional or histological alterations were evident. Therefore, additional experiments were performed to test whether RON could not only prevent, but also reduce glaucomatous neuropathy progression. For this purpose, RON was performed at 6 weeks of treatment with CS, a time point characterized by significant functional (but not histological) alterations. The present results indicate that the delayed treatment (i.e. at 6 weeks of ocular hypertension) also resulted in a significant protection against experimental glaucomatous damage, both at functional and histological level.

Soon after the proposal of RON as a potential treatment modality for CRVO, a debate arose regarding whether the incision of the scleral outlet is a reasonable or dangerous procedure [23], [24]. The potential risk of nerve fiber defects resulting in visual field loss has especially been addressed by many authors [24], [25]. Several surgical complications, such as laceration of the central retinal artery (CRA), further reductions in retinal blood flow, peripapillary retinal detachment from the RON site, optic nerve fiber damage, visual field loss, and focal hemorrhagic pigment epithelium detachment, as well as chorioretinal neovascularization from the RON site were described [16], [26]–[29]. Although the radial incision mode and site, as well as the use of one-blunt-one-sharp-side microvitreoretinal blades specially designed for RON should minimize vessel and nerve fiber injury, these complications cannot be completely ruled out. We have recently shown that in normal rat eyes, RON creates a defect in the lamina cribrosa and surrounding scleral ring of the optic nerve, without affecting the CRV and CRA, neither ocular functions such as VEPs, and pupil light reflex, whereas a transient decrease in ERG was observed in eyes submitted to RON [20]. Moreover, no significant vitreous hemorrhage or other serious complications were found in vehicle- or CS-injected eyes submitted to RON, supporting that at least in rat eyes, RON is a safe procedure.

RON performed at 3 or 6 weeks of ocular hypertension prevented and reduced respectively, the decrease in the ERG a- and b-wave and flash VEP N2-P2 amplitude induced by weekly injections of CS, which indicates that RON not only preserved the retinal function, but also the activity of all cells in the pathway from photoreceptors to visual cortex, including RGCs and their axons.

In addition to RGCs, the GCL is comprised of a number of displaced amacrine cells. NeuN is a DNA-binding protein that identifies most mature neuronal populations, which has been used as a specific marker for RGCs [30], [31], [32]. However, since according to other authors [33], [34], NeuN does not distinguish between RGCs and displaced amacrine cells, two other specific markers of RGCs were included in the present study (Thy-1 and Brn3a). Thy-1 is a surface glycoprotein uniquely expressed in RGCs [35], and Brn3a is a POU domain transcription factor that specifically is expressed in the nuclei of these cells [36]. In the retina from eyes injected with CS and submitted to a sham operation, a significant loss of RGCs was observed, as shown by Thy-1 and Brn3a immunohistochemistry. Similar results were obtained with NeuN immunolabeling, supporting that RON significantly reduced the effect of ocular hypertension on RGC (but not displaced amacrine cell) number. In addition, a significant decrease in the axon number was evident in sham-operated hypertensive eyes, whereas RON significantly preserved ON axon number.

The precise mechanisms responsible for the retinal protection against glaucomatous damage induced by RON remain to be established. For the past 30 years, discussion has focused on how RGC axons are damaged within the lamina cribrosa, and controversy has centered on whether IOP (the mechanical hypothesis) or ONH blood supply (the vascular hypothesis) is responsible for ONH axonal damage in this disease. However, consideration of the anatomy of the lamina cribrosa and peripapillary sclera suggests that the classic mechanical and vascular mechanisms of glaucomatous injury are inseparably intertwined [37]. Moreover, local damage at the lamina has been suggested to account for abnormal axonal transport in glaucoma [38], [39]. Burgoyne et al. [3] have proposed that the ONH is a biomechanical structure; this paradigm assumes that IOP-related stress (force/cross-sectional area) and strain (local deformation of the tissues) are central determinants for the pathophysiology of the ONH tissues and their blood supply, particularly at high levels of IOP [37].

While the original rationale of RON was the relief of increased tissue pressure within the optic nerve that results from occlusion of the CRV, the present results suggest that relaxation of the scleral ring of the prelaminar and laminar regions of the ONH may alleviate the IOP-related connective tissue stress, which in turn, could prevent the onset and reduce glaucomatous neuropathy, presumably by counteracting ONH connective tissue damage, maintaining nutritional and oxygen supply, and axoplasmic transport at increased levels of IOP. In addition, since several lines of evidence support that neuroinflammation and oxidative stress can contribute to RGC death [40], [41], the fact that RON reduced RGC loss could suggest that this surgery could behave as an anti-inflammatory and/or anti-oxidant therapy at retinal level, an hypothesis that deserves to be examined.

A major weakness of the present results relies on the differences between the rat and human eye. Although results from some authors support that the lamina cribrosa in the rat eye is thin and poorly developed [42], the rat eye was shown to be a good model for ophthalmologic studies because its anatomy is similar to that of the human eye. In fact, it was shown that the rat ONH possess an identifiable lamina cribrosa with structural proteins nearly identical to that of the primate [43], [44]. In contrast, the rabbit, chicken, and quail seem to be less adequate models to study the lamina cribrosa, the major problem being the myelinization of the axons penetrating through the sparsely developed lamina cribrosa into the nerve fiber layer of the retina, changing profoundly the situation of cell composition and mechanical reactivity in the ONH region. In addition, the retina from these species is avascular which probably has a major influence on the ONH blood supply too [45]. Moreover, the vascular supply and the localization of the central retinal vessels in rats should be taken into account when comparing findings with the human situation.

Although care must be taken when extrapolating data generated in rodents to humans, our data provide evidence which supports the beneficial effects RON on retinal damage induced by chronic ocular hypertension. Due significance should be given to the fact that the ONH, a very delicate and crucial structure of the eye is being dealt with surgically. However, developments in techniques and technology could increase the margin of safety and efficacy of RON in humans, raising the hope that in the future, benefits of RON against glaucomatous damage could outweigh the risks of this procedure.

## Materials and Methods

### Ethics Statement

All animal procedures were in strict accordance with the ARVO Statement for the Use of Animals in Ophthalmic and Vision Research. The ethic committee of the School of Medicine, University of Buenos Aires (Institutional Committee for the Care and Use of Laboratory Animals, (CICUAL)) approved this study.

### Animals

Male *Wistar* rats (average weight, 200±40 g) were housed in a standard animal room with food and water *ad libitum* under controlled conditions of humidity and temperature (21±2°C), under a 12 h light: 12 h dark lighting schedule (lights on at 07.00 h). A total number of 60 animals were used for the experiments, distributed as follows: for IOP, ERG and VEP assessment: 10 animals bilaterally injected with vehicle (control) and 10 animals bilaterally injected with CS submitted to sham procedure or RON at 3 weeks of intracameral treatment, and the same amount of animals submitted to RON or sham procedure at 6 weeks of intracameral treatment (total amount of animals for these studies: 40). For retina and ON histology: 5 animals bilaterally injected with vehicle (control) and 5 animals bilaterally injected with CS, with RON in one eye and sham procedure in the contralateral eye performed at 3 weeks of intracameral treatment, and the same amount of animals submitted RON in one eye and sham procedure in the contralateral eye at 6 weeks of intracameral treatment (total amount of animals for these studies: 20)

### Intracameral injections

Rats were anesthetized with ketamine hydrochloride (150 mg/kg) and xylazine hydrochloride (2 mg/kg) administered intraperitoneally. With a Hamilton syringe and a 30-gauge needle, under a surgical microscope with coaxial light, 20 µl of CS (Sigma Chemical Co., St. Louis, MO, catalog # C9819, 0.4 g/ml in saline solution) were bilaterally injected into anesthetized rats, and an equal volume of vehicle (saline solution) was bilaterally injected in control rats. Eyes were focused under a surgical microscope (model OMNI MDU XY; Carl Zeiss, Oberkochen, Germany) with coaxial light. The needle moved through the corneoscleral limbus to the anterior chamber with the bevel down. When the tip of the bevel reached the anterior chamber, the liquid progressively increased the chamber's depth, separating the needle from the iris and avoiding needle-lens contact. Applications were made slowly but using a force sufficient to just empty the syringe content (adjusted to 20 µl). Weekly injections were applied at the corneoscleral limbus, beginning from hour 12 and changing the site of the next injection from hour to hour, by rotating the head to achieve better access to the limbus. Injections were performed after application of one drop of 0.5% proparacaine hydrochloride to each eye. Rats showing cataract and animals with phthisis bulbi (less than 5% of animals) were excluded from the experiments. In addition, almost all the animals developed localized corneal edema at the site of the injection that lasted less than 24 h.

### Radial Optic Neurotomy (RON)

Rats were anesthetized as already described. After 3 or 6 weeks of treatment with vehicle or CS, animals were subjected to RON in one eye, whereas the contralateral eye was subjected to a sham procedure. RON was performed as follows: eyes were focused under a binocular Colden surgical microscope with coaxial light for fundus visualization and illumination. Using a 30-gauge dental cartridge needle, a scleral puncture was made at 1 mm of the corneoscleral limbus. A single incision at the nasal hemisphere of the optic disk was performed in the edge of the neuro-retinal ring, cutting an equal part of ON and parapapillary retina, avoiding damage to the central retinal vessels. Care was taken to make the stab radial to the optic disk and parallel to the nerve fiber pattern. The sham operated eyes were submitted to a similar procedure (a scleral puncture was made at 1 mm of the corneoscleral limbus), but without any incision.

### IOP assessment

A tonometer (TonoPen XL; Mentor, Norwell, MA) was used to assess IOP in conscious, unsedated rats, as previously described [1]. IOP determinations were weekly assessed by operators who were blind with respect to the treatment applied to each eye. Animals were wrapped in a small towel and held gently, with one operator holding the animal and another making the readings. Five IOP readings were obtained from each eye by using firm contact with the cornea and omitting readings obtained as the instrument was removed from the eye. Differences among reading were less than 10% (standard error). The mean of these readings was recorded as the IOP for this eye. Mean values from each rat were averaged, and the resultant mean value was used to compute the group mean IOP ± SE. IOP measurements were performed at the same time each day or week (between 11.00 and 12.00 h) to correct for diurnal variations in IOP. IOP was assessed in both eyes of these animals before injections and at 7-day intervals, afterwards.

### Electroretinography

Electroretinographic activity was assessed as previously described [1]. Briefly, after 6 h of dark adaptation, rats were anesthetized under dim red illumination. Phenylephrine hydrochloride and tropicamide were used to dilate the pupils, and the cornea was intermittently irrigated with balanced salt solution to maintain the baseline recording and to prevent keratopathy. Rats were placed facing the stimulus at a distance of 20 cm. All recordings were completed within 20 min, and animals were kept warm during and after the procedure. A reference electrode was placed through the ear, a grounding electrode was attached to the tail, and a gold electrode was placed in contact with the central cornea. A 15 W red light was used to enable accurate electrode placement. This maneuver did not significantly affect dark adaptation and was switched off during the electrophysiological recordings. ERGs were recorded from both eyes simultaneously and ten responses to flashes of unattenuated white light (5 ms, 0.2 Hz) from a photic stimulator (light-emitting diodes) set at maximum brightness (6 cd s/m^2^ without filter) were amplified, filtered (1.5-Hz low-pass filter, 1000 high-pass filter, notch activated) and averaged (Akonic BIO-PC, Argentina). The a-wave was measured as the difference in amplitude between the recording at onset and the trough of the negative deflection and the b-wave amplitude was measured from the trough of the a-wave to the peak of the b-wave. Runs were repeated 3 times with 5 min-intervals to confirm consistency. Mean values from each eye were averaged, and the resultant mean value was used to compute the group means a- and b-wave amplitude ± SEM. The mean peak latencies and peak-to-peak amplitudes of the responses from each group of rats were compared.

### Flash visual evoked potentials

For VEP recording, two stainless steel electrodes were surgically placed 4 mm lateral to the interhemispheric fissure and 5,6 mm behind bregma (active electrode), as previously described [1]. Reference electrodes were placed 2 mm lateral to the midline and 2 mm before bregma. A ground electrode was placed in the animal tail. Both electrodes were isolated and fixed with dental acrylic and the skin was sutured with nylon 5/0. VEPs were assessed 7 days after electrode implantation, as follows: after 6 h of dark adaptation, rats were anaesthetized, pupils were dilated and the cornea was intermittently irrigated as previously described, under dim red illumination. All recordings were completed within 20 min of the induction of anesthesia and animals were kept warm during and after the procedure. Each eye was registered individually, occluding the contralateral eye, and a 70 stimuli average was registered. Eyes were stimulated with unattenuated white light (1 Hz) from a photic stimulator (light-emitting diodes) set at maximum brightness were amplified, filtered (0.5-Hz low-pass filter, 100 high-pass filter, notch activated) and averaged (Akonic BIO-PC, Akonic, Argentina). The amplitude between the N2 deflection and the P2 peak was assessed, and the N2 latency was measured from de onset to the second negative peak.

### Histological analysis

Eyes were enucleated after anesthetic overdose and immersed immediately in a fixative containing 4% paraformaldehyde in 0.1 M phosphate buffer (pH 7.2) for 1 h. The nictitans membrane was maintained in each eye to facilitate orientation. The cornea and lens were carefully removed, and the posterior portions were fixed for an additional 12 h- period in the same fixative. A cross section of the optic nerve from vehicle and CS-treated eyes was removed 1.5 mm posterior to the globe and postfixed in 1% osmium tetroxide in phosphate buffer. Nerves were processed into epoxy resin, sectioned at 1 µm, and stained with 1% toluidine blue. Eyecups were dehydrated in an alcohol series, and embedded in paraffin. Sections (5 µm thick) were cut along the horizontal meridian through the ONH and stained with H&E.

### Immunohistochemical studies

Antigen retrieval was performed by heating (90°C) slices for 30 min in citrate buffer and then preincubated with 2% normal horse serum, 0.1% bovine serum albumin, and 0.4% Triton X-100 in 0.01 M phosphate-buffered saline for 1 h. The sections were then incubated overnight at 4°C with a mouse monoclonal anti-NeuN antibody (1∶120; Millipore, Temecula, CA, USA), or a goat anti-Brn3a (1∶500 Millipore, Temecula, CA, USA) antibody. An anti-mouse and anti-goat secondary antibody conjugated to Alexa Fluor 568 (1∶500; Molecular Probes, Grand Island, NY, USA) were used. After immunostaining, the sections were mounted with antifade medium with the fluorescent nuclear stain DAPI (Vector Laboratories, Burlingame, CA, USA). For Thy-1 level assessment, endogenous peroxidase activity was blocked with 0.3% H_2_O_2_ in phosphate- buffered saline (PBS) for 20 min, and the sections were then incubated overnight at 4°C with a mouse monoclonal anti-Thy-1 antibody (1∶500 Millipore, Temecula, CA, USA). Thy-1 positive signal was developed with the labeled streptavidin-biotin (LSAB2® System HRP Dakocytomation, Dako, Carpinteria, CA, USA) reagent kit, according to manufacturer's instructions. Some sections were treated without the primary antibodies to confirm specificity. An Olympus BX50 microscope (Olympus, Tokyo, Japan) was used for microscopic observations. Comparative digital images from different samples were grabbed using identical time exposition, brightness, and contrast settings.

### Image analysis

Microscopic images were digitally captured with a Nikon Eclipse E400 microscope (illumination: 6-V halogen lamp, 20 W, equipped with a stabilized light source) attached to a digital camera (Coolpix s10; Nikon). The digitalized images were transferred to a Scion Image for Windows analysis system (Scion Corporation Beta 4.0.2).

Retinal morphometry was evaluated as previously described [1]. Three sections were randomly selected from each eye. Nine microscopic images at 1 mm from the temporal edge of the optic disc were digitally analyzed. The light microscope was adjusted to level 4 and a 40× CF E achromat objective was used. The thickness (in µm) of the IPL, INL, OPL, ONL, and total retina was measured. The number of cells in the GCL was expressed as cells per 100 µm. For each eye, results obtained from three separate sections were averaged and the mean of 5 eyes was recorded as the representative value for each group. The morphometric analysis was performed by observers masked to the protocol used in each eye.

### Optic nerve morphometry

ON axon counting was performed as previously described [1]. Images were captured with a 100× achromat objective from 5 spaced nerve regions, converted to 8-bits grey scale and a manual threshold value, first determined by visual examination, was constantly applied. Finally, images were converted to a binary form. The number of axons counted in 5 images from each nerve was approximately 10% of the total optic nerve area. The counting process was performed by observers masked to the protocol used in each nerve.

### Statistical analysis

Statistical analysis of results was made by a two-way analysis of variance (ANOVA) followed by Tukey's test, as stated.
